# Audit report of physical health examination and baseline investigations on high dependency unit (HDU) and acute ward, Nepean Mental Health Centre, Sydney, Australia

**DOI:** 10.1192/bjo.2021.863

**Published:** 2021-06-18

**Authors:** Adil Jawad, Kristof Mikes-Liu, Christopher Mah, Amgad Elmakki, Riffat Fatima

**Affiliations:** Nepean Hospital, Mental Health Centre

## Abstract

All patients on High Dependency Unit (HDU) and Acute Ward, Mental Health Centre, Nepean Hospital, were included in a cross-sectional audit on 22nd January 2020. Out of a total of 43 patients admitted on both these wards, 88.4% had baseline blood tests done, but almost half did not have baseline ECG done and 1/3rd did not have a physical examination done. The physical examination on admission on these wards is better than in 2017 & 2018 when half and more than 1/3rd respectively did not have physical examination done.

